# Implementing Carbon Capture and Storage in the United Kingdom: Estimating Willingness to Pay through a Contingent Valuation Survey

**DOI:** 10.1007/s00267-025-02174-6

**Published:** 2025-04-22

**Authors:** Tanisha Waring, Alberto Longo

**Affiliations:** 1 School of Biological Sciences, Gibson Institute for Land, Food & Environment, IGFS, Queen’s University Belfast, 19 Chlorine Gardens, Belfast, BT9 5DL Northern Ireland UK; 2 Present Address: Department of Agriculture and Forest Sciences, Rettorato, Via S.M. in Gradi n.4, 01100 Viterbo, Italy

**Keywords:** Contingent valuation, Willingness to pay, Carbon capture and storage, Climate change, Mitigation, Public perceptions

## Abstract

Carbon capture and storage (CCS) is a technology implemented to reduce emissions in the power and industrial sectors by capturing carbon dioxide (CO_2_) before it is released into the atmosphere. Emerging technologies like CCS are often unfamiliar to the public and can be misinterpreted when publics are not involved in the decision-making process, leading to opposition and barriers to deployment. Engaging the public on policy decisions can prevent such obstacles from hampering implementation. This study aims to elicit preferences and estimate the distribution of willingness to pay (WTP) for carbon capture and storage in the United Kingdom (UK). We employ the contingent valuation method with double bounded dichotomous choice format, administering an online survey to 1033 individuals from the UK. Interval regression analysis is applied to estimate mean WTP. Our findings indicate that public attitudes towards CCS in the UK tend to be relatively positive. Mean WTP for the implementation of CCS is £95.50. We find that environmental attitudes more than socio-demographic characteristics are significant factors in WTP decisions for CCS. Public acceptance is critical to ensure appropriate steps are taken to move CCS projects and policy forward and prevent further delay in tackling emissions in the energy sector.

## Introduction

Carbon capture and storage (CCS) refers to technology applied to large-scale carbon dioxide (CO_2_) emitters such as power plants and other industrial facilities to capture CO_2_ before it can be released into the atmosphere. The CO_2_ is then transported to a suitable storage site such as a depleted oil or gas field or saline aquifer. Here the CO_2_ remains sealed from the atmosphere, thereby making CCS a climate mitigation measure (Haszeldine 2009; Kraeusel and Möst 2012). CCS is often considered a ‘bridging technology’, acting as an interim to a more sustainable energy system. The technology enables the continued use of fossil fuels to meet ever-increasing energy demand, which renewable sources are not presently equipped to handle, while simultaneously reducing CO_2_ emissions (Vergragt et al. 2011). CCS is increasingly recognized as a feasible, and even necessary, option to tackle climate change. Almost all projections indicate that a 2 °C, let alone 1.5 °C, target can only be achieved through large-scale carbon capture interventions (Braun et al. 2018). The present study investigates public perceptions of CCS in the United Kingdom where stringent emissions targets have been committed to, namely bringing all GHG emissions to net zero by 2050 (Burnett et al. 2024). Such an ambitious target is likely to require the assistance of carbon capture technologies if it is to be achieved.

Large-scale deployment of CCS is contingent not only on policy and technical feasibility, but also on public acceptability. With decades of technical experience across the entire value chain, it is clear that it is not a lack of technical expertise that inhibits the deployment of CCS technology (Bui et al. 2018). Rather, as is often the case with emerging technologies, barriers to CCS deployment stem from public opposition. CCS projects are unlikely to move forward in a democratic society without considerable public support (Boyd et al. 2017). Public opposition has proven to be a key obstacle to the development of new energy infrastructure including oil refineries, nuclear power plants, and even wind farms (Fleishman et al. 2010). Perlaviciute et al. (2018) argue that public acceptability is at the heart of a sustainable energy transition but is often addressed too late and should be incorporated into the planning process for energy projects from the beginning. They define public acceptability as a broad concept referring to people’s general evaluation of energy projects, or simply, the extent to which they favour or disapprove of a particular energy project (Perlaviciute et al. 2018:50).

This study aims to examine public perceptions and willingness to pay for CCS in the UK using a contingent valuation (CV) survey. This approach enables stakeholders to discern i) the extent of support for CCS in the UK, ii) if there is any willingness to pay for CCS at all, and iii) if so, to what extent. A secondary objective of this study is to explore how WTP varies across the population and provide insights into how CCS is perceived by different groups in society. This includes socio-demographic characteristics such as gender, age, education and income as well as environmental attitudes.

The paper is structured as follows. Section 2 presents findings from previous research on public acceptance of CCS. Section 3 outlines the CV methodology employed in the study. Section 4 presents the analysis of the survey data and discusses these results in detail, concluding with relevant policy and research implications.

## Previous Research on Public Perceptions of Carbon Capture and Storage

Social acceptance of CCS was first explored around twenty years ago and has gained traction since, particularly in the past decade. These studies have undoubtedly added to our understanding of perceptions of the technology. However, each study has been applied in a different context, involving different actors (stakeholders, experts, and the public) and using a variety of approaches to quantify these perceptions. Consequently, it is difficult to compare results or find consistent outcomes. In terms of how the public perceive CCS, a number of trends have emerged in the literature, however, findings are inconsistent and often contradictory. The most consistent results from studies on perceptions of CCS indicate that i) awareness of CCS is low among the public, and ii) that support for the technology is often conditional at best and context-dependent.

The first major finding in the literature is that the public do not tend to be aware of what CCS entails (de Best-Waldhober et al. 2009; Oltra et al. 2010; Arning et al. 2019). Despite the issue of lack of accessible information on CCS decreasing over recent years, CCS remains relatively unknown to most lay public (Ashworth et al. 2015). In China, Chen et al. (2015) observed a lack of scientific knowledge about CCS among the public, and among respondents in a survey conducted by Duan (2010), only 24% knew about CCS technology. Similar results were found in Indiana, USA (Carley et al. 2012), Québec, Canada (Moutenet et al. 2012) and in France (Ha-Duong et al. 2009) where only 6% of respondents were able to provide a satisfactory definition of the technology. Familiarity with CCS is higher by comparison in the Netherlands, likely because of the notoriety of the failed Barendrecht carbon capture project (Upham and Roberts 2011). In an international comparison of public attitudes towards CCS technologies, Reiner et al. (2006) observed low to very low levels of awareness, and in some countries even those who express familiarity do not appear to understand what problem CCS aims to address. However, it is important to note that while there is broad agreement in the literature that the public remain largely unaware of CCS technologies, the link between awareness and acceptance of energy projects has not been proven (Cohen et al. 2014). While some studies find positive correlation between awareness and acceptance (Duan 2010), others indicate that providing information with the aim of increasing respondents’ knowledge of CCS has a negative effect or no effect at all (Shackley et al. 2007; Sharp et al. 2009).

In terms of how public acceptance is often context-dependent, the literature to date highlights a number of underlying factors at play in shaping opinions. These include regional variations, characteristics of the community and socio-political context, risk perception, trust in industry, government and other stakeholders, and the level of public engagement. One example can be observed between public perceptions in Canada and Switzerland. Canadian publics are more accepting of CCS compared to Swiss publics, perhaps because of the difference in energy strategies between the two countries (Whitmarsh et al. 2019). In Canada there is greater appreciation of fossil fuels as drivers of economic growth. In contrast, Switzerland has no power plants generating energy with fossil fuels and CCS would be linked to the phase out of nuclear energy. This would involve using natural gas to generate power, rendering CCS for the purpose of maintaining current emissions which fuels concerns about sustainability (L’Orange Seigo et al. 2014). Therefore, energy and economic strategies can influence perceptions of CCS implementation. Perception of risk also often influences a person’s decisions and behaviour (Siegrist et al. 2005). With regards to CCS, the public may be concerned about the potential for induced seismicity (Wong-Parodi and Ray 2009) as well as potential for CO_2_ leaks (Bradbury et al. 2011) and the impact this can have on human health and groundwater (Feldpausch-Parker 2010) or local wildlife (Boyd 2016).

### Willingness to Pay for Carbon Capture and Storage

In this study, as well as investigating public attitudes towards CCS, we also estimate the distribution of willingness to pay for the implementation of the technology in the UK. Following Kraeusel and Möst (2012), we see the determination of WTP as an additional measure to quantify the acceptance of CCS rather than as a rationale for a tax to implement CCS. The principle of social acceptance research is to conclude from current attitude to future behaviour (Kraeusel and Möst 2012). Assessing the WTP of the general public for climate change mitigation programs enables governments to understand how much taxpayers are willing to support the implementation of such programs in the future (Longo et al. 2012). Mitigating and adapting to climate change is a costly endeavour and households will likely be presented with higher prices, or taxes, or a combination of the two in order to cover the costs of responding to climate change. Consequently, estimating the WTP for mitigating the effects of climate change is useful to determine the mitigation targets that governments should undertake with the support of the taxpayers (Layton and Brown 2000) as well as serving as a tool for designing adapted marketing and promotion strategies (Kraeusel and Möst 2012).

Limited research has been conducted into WTP for CCS. However, in a choice experiment by Kraeusel and Möst (2012), German consumers expressed a neutral attitude towards CCS but did not express much WTP. Respondents were willing to pay a €1.54 monthly price premium for a 10% increase in CCS power. In comparison, they were willing to pay €10.85/month for a 10% increase in renewable power, indicating that levels of public support are much higher for renewables than for CCS. Similar findings are reported by Itaoka et al. (2017) in a comparative study of WTP for renewables, nuclear, and thermal power generation with CCS in Japan. Respondents here are willing to pay 11 yen per 1% increase in renewables, 14 yen per 1% decrease in nuclear, and 4 yen per 1% increase in CCS. The findings from these studies indicate that public opinion on CCS is neutral at best and consumers are willing to pay less for its implementation in comparison to renewables. While public opinion of CCS in the UK has been analysed before (Shackley et al. 2004; Whitmarsh et al. 2019; Cox et al. 2020), WTP has not been incorporated into these studies and thus WTP for the technology here is unknown.

## Methodology

### Study Area

The UK is particularly well placed to deploy CCS at commercial scale given the industry experience gained through oil and gas development as well as the significant storage potential in the North Sea in saline aquifer formations and depleted hydrocarbon fields. There is also potential for industry ‘clustering’ in the UK whereby multiple carbon emitters (power stations, industrial facilities) use shared transport and storage infrastructure. CCS projects and policies in the UK have consisted of false starts over the past two decades. CCS was first recognised as “a promising way forward” in the 2003 Energy White Paper (Great Britain. Department of Trade and Industry 2003). Since then a number of policies have been put forward, but CCS is yet to be deployed at scale in the UK.

### Sampling Design

We conducted an online contingent valuation survey with a representative sample of the UK population (*n* = 1033). Participants were recruited through a third-party market research company, CheckMarket and the survey built through their online platform. The sampling design was stratified by region (England, Scotland, Wales and Northern Ireland), age and gender and the sample drawn randomly from the UK population. Participants were not offered an incentive by the research team for completing the survey.

### Contingent Valuation Method

We employed a contingent valuation approach to our study given that CCS is not currently implemented at scale in the UK and there is no real world market in which observations can be made. Contingent valuation is a survey-based stated preference approach which directly elicits individuals’ WTP for a particular non-market good (Champ 2003). It is so-called because the valuation estimate obtained is said to be ‘contingent’ on the hypothetical market presented in the survey (Carson and Hanemann 2003). The CV survey was designed in accordance with best possible practice (Arrow et al. 1993; Hanemann et al. 1991; Mitchell and Carson 1989). The first section of the survey collected data on respondents’ knowledge of and attitudes toward climate change and the environment. In order to explore how environmental attitudes influence perceptions and WTP towards CCS, we incorporate environmental identity as well as environmental values as potential explanatory variables using a reduced version of the New Environmental Paradigm (NEP) scale (Dunlap and Van Liere 1978). Respondents are asked to indicate the extent to which they agree with statements about the environment. These statements, adapted from previous research (Cook et al. 2002; Whitmarsh and O’Neill 2010), measure environmental identity (“I consider myself as someone who is concerned about the environment”) as well as environmental values (“economic development is more important that environmental protection”). Environmental identity and environmental values are assumed to be different in this study given that individuals may consider themselves as an environmentalist, but their values may not necessarily reflect this. In addition, respondents are asked if they are part of an environmental organization such as the National Trust or Friends of the Earth to gain further insight into their attitudes towards environmental protection. The second section comprised of the hypothetical CV scenario and value elicitation questions (discussed in detail below). Following the values elicitation questions, respondents were presented with a number of potential reasons for making their decision and asked to indicate which of these applied to their own personal WTP decision. The final section of the survey gathered respondents’ socio-economic characteristics including age, gender, education, employment, and income.

### CV Scenario and Payment Vehicle

The CV scenario is intended to give respondents a clear picture of the good they are being asked to value (Portney 1994). It is therefore critically important for eliciting reliable WTP values. If respondents misunderstand or do not fully believe the CV scenario, their responses will reflect this, resulting in implications for the data. In this study, respondents were first provided with background information on CCS including what it is, why it is being considered, and how the technology works, before being issued with an introductory statement to the hypothetical scenario. Here they are asked to assume that the implementation of CCS in the UK can only be achieved by raising household taxes. The payment vehicle must also be given significant consideration to ensure it adds to the credibility of the CV scenario (Pearce et al. 2002; Mitchell and Carson 1989; Carson and Hanemann 2003) and therefore must be plausible and coercive. For this study, a one-off tax was selected as it is familiar to the sample population and has higher incentive compatibility compared to voluntary mechanisms (Einarsdottir et al. 2019). While a one-time payment can produce more conservative values, owing to the lack of opportunity to spread payments over time, a one-time payment is appropriate when providing a good or service is a one-time event (Carson 2000). Given that this study is seeking a WTP value for the initial implementation of CCS in the UK, a one-time lump sum payment was considered appropriate. An advanced disclosure approach was applied to the CV scenario as suggested by Bateman (2002) whereby respondents are informed of what will be expected of them before they perform the tasks.

### Elicitation of WTP and Statistical Model

Following recommendations in the literature (Arrow et al. 1993; Bateman, 2002), the survey used the double-bounded dichotomous choice (DBDC) format to elicit respondents’ WTP. This is an extension of the single-bounded dichotomous choice (SBDC) format where respondents are asked ‘Would you pay £B for X?’ for a range of bid amounts. The DBDC format involves another round of bids conditional on responses to the first bid and therefore provides more information on WTP (Hanemann 1985; Carson, Hanemann and Mitchell 1986). As a result, the statistical efficiency of the model is improved (Kanninen 1993). In our survey a range of starting bids were randomly assigned to respondents to reduce starting point bias. These bids were selected following a focus group (*n* = 10) and pilot study (*n* = 208) in January and May of 2020 respectively. The pilot study was conducted through CheckMarket and with a representative sample of the UK population. Within the focus group we asked an open-ended question for participants to indicate an amount of money they would pay towards the implementation of CCS. Based on this, participants in the pilot study were presented with starting bids of £5, £10, £25, £50 and £100. Following recommendations in the literature (Lopez-Feldman 2012), we sought to explore if as the bid amount increased, the proportion of individuals accepting the bids decreased. The pilot indicated that 55% were willing to pay the middle bid. However, only 68% accepted the lower bid of £5 and a comparatively higher number of respondents accepted the higher bid of £100 (38%). Consequently, the bid amounts were revised and increased to £5, £15, £50, £100 and £250.

Respondents were presented with one of five initial bids (B_1_) and distributed equally across these categories. If the respondent accepted the initial bid, the second bid value (B_2_) was increased, while if the respondent rejected the initial bid, B_2_ was decreased. The bid design employed in this study is outlined in Table 1.

**Table 1:** survey bid design

	Amount in 1^st^ bid (B_1_)				
	£5	£15	£50	£100	£250
B_2_ if ‘no’ to B_1_	£2	£5	£15	£50	£100
B_2_ if ‘yes’ to B_1_	£15	£50	£100	£250	£500

Willingness to pay is assumed to be a linear function of economic factors such as income, and demographic and attitudinal variables. These are assumed to be included in a vector X. Following the random utility framework, the individual’s WTP is a random variable with a given cumulative distribution function (cdf), denoted by G (Ci; θ) where θ represents the parameters of the distribution, which are to be estimated on the basis of the responses to the CV survey, and Ci is the individual’s true maximum WTP for the implementation of CCS. WTP is thus specified as (Haab and McConnell 2002) Eq. (1), where WTP is the WTP of individual i, μ_i_ is the mean WTP where μ_i_ = X_i_β with β being the vector of the coefficients to be estimated, and ε_i_ is the error term.

1 $${{WTP}}_{i}={\mu }_{i}+{\varepsilon }_{i}$$

In the DBDC model, respondents are divided into four groups, determined by their responses to the bid amounts. For each individual i with WTP, a binary-valued indicator variable represents whether the individual belongs to one of the four groups set out in Eqs. (2)–(5).

2 $${{\rm{I}}}_{{\rm{i}}}^{{\rm{YY}}}=1{\rm{ifWTP}}\ge {{\rm{t}}}_{{\rm{i}}}^{{\rm{U}}}(0{\rm{if\; otherwise}})$$

3 $${{\rm{I}}}_{{\rm{i}}}^{{\rm{YN}}}=1{\rm{if}}{{\rm{t}}}_{{\rm{i}}}\le {\rm{WTP}} < {{\rm{t}}}_{{\rm{i}}}^{{\rm{U}}}(0{\rm{if\; otherwise}})$$

4 $${{\rm{I}}}_{{\rm{i}}}^{{\rm{NY}}}=1{\rm{if}}{{\rm{t}}}_{{\rm{i}}}^{{\rm{L}}}\le {\rm{WTP}} < {{\rm{t}}}_{{\rm{i}}}(0{\rm{if\; otherwise}})$$

5 $${{\rm{I}}}_{{\rm{i}}}^{{\rm{NN}}}=1{\rm{if}}0 < {\rm{WTP}} < {{\rm{t}}}_{{\rm{i}}}^{{\rm{L}}}(0{\rm{if\; otherwise}})$$

where:${{\rm{t}}}_{{\rm{i}}}$ denotes the initial bid${{\rm{t}}}_{{\rm{i}}}^{{\rm{U}}}$ ${\rm{and}}$ ${{\rm{t}}}_{{\rm{i}}}^{{\rm{L}}}$ denote the second bids (upper and lower respectively)${{\rm{I}}}_{{\rm{i}}}^{{\rm{YY}}}$ a participant accepting both bids${{\rm{I}}}_{{\rm{i}}}^{{\rm{YN}}}$ a participant accepting the first bid and rejecting the second bid${{\rm{I}}}_{{\rm{i}}}^{{\rm{NY}}}$ a participant rejecting the first bid and accepting the second bid${{\rm{I}}}_{{\rm{i}}}^{{\rm{NN}}}$ a participant rejecting both bids.

Assuming that the cdf is a logistic distribution, the log-likelihood of the model is represented by Eq. (6), where yy, yn, ny, nn represent the responses of the individual and N_yy_, N_yn_, N_ny_, N_nn_ correspond to the number of occurrences of each response type.

6 $${LL}=\sum _{i=1}^{{N}_{{yy}}}{yylog}\left({prob}\left({yy}\right)\right)+\sum _{i=1}^{{N}_{{yn}}}{yn}\mathrm{log}\left({prob}\left({yn}\right)\right)+\sum _{i=1}^{{N}_{{ny}}}{ny}\mathrm{log}\left({prob}\left({ny}\right)\right)+\sum _{i=1}^{{N}_{{nn}}}{nnlog}({prob}\left({nn}\right))$$

## Results

### Descriptive Statistics

The survey was administered to 1248 individuals from across the UK. The sampling design was stratified by region, age and gender to ensure the sample accurately represented the UK population. Following data cleaning and removal of protest responses, the final sample size was 1033 respondents. Table 2 presents descriptive statistics of the sample. The percentage of females in our sample is 51.5%. 33.3% of the respondents are aged 55 or over. Within our sample, 45.5% have attained a tertiary level of education, which is defined as at least a foundation degree or equivalent and 56.63% are employed full-time. We questioned respondents on their knowledge of climate change and 80.15% described themselves as ‘fairly’ or ‘very well’ informed. Less than half of the sample (47.24%) stated they had ‘high’ or ‘very high’ concern about climate change. 46.27% of the sample stated they had not heard of and therefore did not know what CCS is. However, 60.79% felt that CCS would be an effective measure for tackling climate change and 62.05% were in favour of the technology being deployed.

**Table 2:** Descriptive statistics

Variable (acronym used in regressions)	Sample percentage
Female (female)	51.50
*Age*	
18–24	11.91
25–34	18.39
35–44	17.42
45–54	18.97
55–64	14.91
65–74	13.36
75+	5.03
Younger respondents aged 18–34 (young)	30.30
Mature respondents aged 55+ (mature)	33.30
*Income*	
<£10,000	9.00
£10,000–£29,999	40.66
£30,000–£59,999	36.21
£60,000–£99,999	11.62
£100,000–£149,999	1.65
>£150,000	0.87
Income below £30,000 (lowincome)	49.66
Live in England (england)	81.10
Live in a town or city (town_city)	82.77
Are married or co-habiting (married)	54.50
Have a tertiary level education (tertiary)	45.50
Are employed full-time, part-time or self-employed(employed)	56.63
Are part of an environmental organisation (envorg)	16.55
Have a strong environmental identity (envidentity)	80.25
Have strong environmental values (envvalue)	47.05
Feel they are well informed on climate change (informed)	80.15
Express high concern about climate change (highconcern)	47.24
Feel CCS would be an effective climate mitigation measure (effective)	60.79
Are in favour of CCS being implemented (favour)	62.05
Are opposed to a CCS site near to their home or community (opposehome)	33.69
Express support for CCS but switch to opposition when a site is proposed near to their home or community (notnear)	14.33
Are unfamiliar with CCS technology (ccs_notheard)	46.27

### Responses to Payment Questions

In a dichotomous choice CV study, the probability of a ‘yes’ response to the payment question should decrease monotonically as the bid amount increases (Haab and McConnell 2002; Alberini et al. 2005). Table 3 shows that 85.05% of respondents who received £5 as their first bid were willing to pay this amount. The proportion of respondents agreeing to the payments decreases as the bid amount increases with only 34.39% of those presented with £250 as their starting bid accepting this amount. Table 4 presents the responses to the initial and follow up bid amounts, outlining the number and percentage of respondents falling into the four answer categories: “yes-yes” (YY), “yes-no” (YN), “no-yes” (NY), and “no-no” (NN). 31.66% of respondents answered “yes-yes”.

**Table 3:** Distribution of yes responses to initial bids (%)

	£5	£15	£50	£100	£250
No	13.95	32.73	40.65	51.79	65.61
Yes	86.05	67.27	59.35	48.21	34.39

**Table 4:** Distribution of responses to the DBDC questions (%)

B_1_	YY		YN		NY		NN		Total	
	*n*	%	*n*	%	*n*	%	*n*	%	*n*	%
£5	136	13.17	49	4.74	11	1.06	19	1.84	215	20.81
£15	80	7.74	68	6.58	29	2.81	43	4.16	220	21.30
£50	73	7.07	54	5.23	41	3.97	46	4.45	214	20.72
£100	20	1.94	74	7.16	30	2.90	71	6.87	195	18.88
£250	18	1.74	47	4.55	24	2.32	100	9.68	189	18.30
Total	327	31.66	292	28.27	135	13.07	279	27.01	1033	100.00

### Statistical Models of the WTP Responses

Table 5 presents several specifications of the WTP regression based on various subsets of the explanatory variables. In Model 1, we use only socio-economic variables as regressors. Only two variables are statistically significant (lowincome and envorg), indicating that individuals with membership to an environmental organization have a higher WTP for CCS than those who are not members of such groups, and as expected, individuals with a lower income have a lower WTP compared to those with a higher income.

**Table 5:** WTP regression results

	Model 1		Model 2		Model 3		Model 4	
	Coeff.	*p*-value	Coeff.	*p*-value	Coeff.	*p*-value	Coeff.	*p*-value
Intercept	1.238 (0.277)	8 ×10^−6***^	0.209 (0.215)	0.332	0.188 (0.357)	0.599	0.506 (0.206)	0.014**
Female	−0.102 (0.121)	0.402			0.068 (0.135)	0.617		
Young	−0.061 (0.151)	0.687			−0.024 (0.159)	0.880		
Mature	0.138 (0.163)	0.398			0.205 (0.174)	0.239		
England	0.064 (0.154)	0.681			0.131 (0.159)	0.412		
Town_city	0.158 (0.162)	0.329			0.184 (0.172)	0.284		
Married	−0.101 (0.128)	0.430			0.001 (0.135)	0.994		
Tertiary	0.176 (0.125)	0.158			−0.002 (0.133)	0.986		
Employed	−0.012 (0.138)	0.933			−0.023 (0.146)	0.872		
Lowincome	−0.713 (0.131)	2.2 ×10^−16^***			−0.725 (0.138)	2.2 ×10^−16^***	−0.689 (0.126)	2.2 ×10^−16^***
Envorg	0.636 (0.175)	0.000***			0.398 (0.183)	0.029**	0.450 (0.179)	0.012**
Envidentity			0.574 (0.171)	0.001***	0.533 (0.174)	0.002**	0.596 (0.162)	0.000***
Envvalue			0.571 (0.133)	1.6 ×10^−5^***	0.591 (0.135)	1.2 ×10^−5^***	0.620 (0.130)	2 ×10^−6^***
Informed			−0.083 (0.164)	0.614	−0.036 (0.167)	0.830		
Highconcern			0.227 (0.139)	0.103	0.208 (0.143)	0.146		
Ccs_notheard			−0.316 (0.129)	0.014**	−0.239 (0.138)	0.082*	−0.267 (0.128)	0.037**
Effective			0.506 (0.149)	0.001***	0.534 (0.154)	0.001***	0.528 (0.151)	0.000***
Favour			0.506 (0.149)	0.001***	0.471 (0.152)	0.002**	0.485 (0.149)	0.001***
Opposehome			−1.016 (0.175)	2.2 ×10^−16^***	−1.041 (0.178)	2.2 ×10^−16^***	−1.019 (0.176)	2.2 ×10^−16^***
Notnear			0.781 (0.217)	0.000***	0.794 (0.221)	0.000***	0.754 (0.219)	0.001***
Observations	1033		10330		1033		1033	
Log-likelihood	−1579.097		−1510.194		−1488.619		−1491.697	
AIC	3182.193		3042.387		3019.237		3005.394	
BIC	3241.476		3096.729		3122.982		3059.736	

Standard error in parentheses

***p < 0.01; **p < 0.05; *p < 0.1

Model 2 explores the effects of attitudinal variables on WTP. In Model 3, we combined the two sets of explanatory variables- socio-economic and attitudinal variables. Model 4, the final model, includes only the statistically significant variables (lowincome, envorg, envidentity, envvalue, ccs_notheard, effective, favour, opposehome, and notnear). Comparing the models for goodness of fit (AIC and BIC), Model 4 outperforms the other models. However, Model 3 is a more comprehensive model and the higher BIC value could be because the BIC penalizes model complexity more heavily (Bishop 2006).

Following the elicitation of respondents’ WTP, we asked a debriefing questions pertaining to the reasons underlying these choices as recommended by the NOAA Panel on contingent valuation (Arrow et al. 1993). The options presented in the survey are presented in Table 6 along with the proportion of respondents who felt these reasons shaped their WTP decisions. A significant proportion of the respondents stated that their concern about climate change influenced their WTP choices (64.38%). 33.69% of respondents also felt that they have a personal responsibility to tackle climate change. Also of interest is that only 15% of respondents felt that the technology would discourage mitigation efforts. This so-called ‘mitigation deterrence’ or ‘moral hazard’ (McLaren et al. 2013; Campbell-Arvai et al. 2017; Lutzke and Árvai 2021) effect is a significant concern for researchers and environmentalists and therefore it is interesting that this sample of the public do not view it with the same apprehension. In addition, around a third (32.82%) of the sample expressed their concern for energy security as an influencing factor in their decision.

**Table 6:** Responses to debriefing questions on reasons for WTP decisions

	*n*	%
I am concerned about climate change	665	64.38
I find the proposed technology realistic	270	26.14
I feel I have a personal responsibility to tackle climate change	348	33.69
I can afford the costs of the tax	389	37.66
I am concerned about the future uninterrupted availability of energy resources at an affordable price	339	32.82
The government should pay for the technology, not citizens	174	16.84
I cannot afford the costs of the tax	157	15.20
I believe that the technology will discourage efforts to reduce GHG emissions	155	15.00

### Mean Willingness to Pay and Socio-demographics

Building on the regression model (Model 3), the mean results were analysed. This was completed for the statistically significant variables as well as age and gender. These results are presented in Table 7 below. Overall, mean WTP for the whole sample was £95.49. While not statistically significant, we found that females have a higher WTP than males with a mean WTP of £97.11 compared to £93.79. In addition, respondents aged 55 or over had a higher mean WTP (£102.29) compared to younger respondents. Respondents aged 18–34 had a mean WTP of £94.68. While WTP for CCS has not been widely studied in the literature, public perceptions of the technology have been, with which we can draw comparisons on the underlying factors of public support for the technology. Duan (2010) conducted a study on public perceptions of CCS as a measure to reduce CO_2_ emissions in China. They find that older respondents were less likely to support CCS than younger respondents. In contrast, we find that older respondents in our sample express more support for CCS as reflected in their higher WTP. Perdan et al. (2017) found in their study of UK perceptions of carbon capture and utilisation (CCU) that older respondents were less concerned about the location of CCU facilities in their area. Further, respondents aged 65 or over were most in favour of the implementation of CCU. However, CCU and CCS are two related yet distinct mitigation measures and significant differences between the perception and acceptance of each have been found (Arning et al. 2019). Therefore, caution should be taken in comparing these results with those found in the present study. Older participants may also have a higher income than younger respondents which could also account for the unexpected higher WTP value found in our sample.

**Table 7:** Mean WTP and 95% CI for socio-economic subgroups in the population

Group	WTP (£)	95% CI	
		Lower bound (£)	Upper bound (£)
Overall WTP	95.50	85.32	105.81
Gender			
Males	93.79	82.83	105.81
Females	97.11	85.41	109.22
Age			
Young (18–34)	94.68	81.27	110.28
Mature (55+)	102.29	82.83	105.81
Income			
Low income	78.37	69.25	87.98
High income	113.79	100.03	128.59
Environmental organisation			
Member	112.31	93.13	132.08
Non-member	92.29	82.42	102.59
Environmental identity			
Agree	100.71	90.62	111.56
Disagree	75.59	62.39	88.29
Environmental values			
Disagree	82.31	72.39	93.06
Agree	111.32	98.88	124.38
Familiarity with CCS			
Not heard of CCS	89.27	78.68	101.32
Heard of CCS	101.00	88.83	114.32
Effectiveness of CCS			
Effective	105.98	94.05	119.24
Ineffective	80.18	69.31	92.48
NUMBY reaction			
Present	130.99	109.45	153.05
Not present	89.99	80.41	101.33

As expected under the assumptions of economic theory, respondents with a lower household income of less than £30,000 per year before tax had a lower mean WTP of £78.37. Those who did not fall into this category and had a higher level of income had a mean WTP of £113.79. Other studies have also determined that income is a common socio-demographic factor underlying WTP for climate mitigation in the energy sector (Alberini et al. 2018; Bergmann et al. 2008; Guo et al. 2014; Lee and Heo 2016). Individual income determines individual WTP (Baumgärtner et al. 2017) with individuals with a higher level of income expressing greater WTP (Krupnick and Adamowicz 2007), as an individual with a lower income may be unable to afford this additional cost, regardless of whether they would like to support CCS or not.

### Mean Willingness to Pay and Knowledge

Mean WTP was calculated for respondents who had not heard of CCS and did not know what the technology is. The mean of these respondents was £89.27, which is much lower than the mean of those who have heard of the term CCS, which was £101.00. Once informed of what CCS entails, respondents were asked how effective they felt it would be in tackling climate change. Those who felt it would indeed be effective had a higher WTP (£105.98) than those who did not feel it would be effective (£80.18). These findings are supported by Duan (2010) who found a positive correlation between knowledge of CCS and support for its implementation in China. Fleishman et al. (2010) further demonstrate that acceptance of, and even preference for CCS is more likely when individuals are more familiar with the technology. However, Reiner et al. (2013) find that despite an increase in awareness and understanding of CCS between 2004 and 2013, knowledge of what environmental problem CCS aims to address remained poor. Further, general support diminished when respondents were asked if they were willing to support the £1 billion capital support for CCS demonstration in the UK (from 42% to 31%).

### Mean Willingness to Pay and Location

We find that respondents who oppose a CCS site being located near to their home or community are willing to pay less to support the implementation of the technology. This is not an unexpected result given that projects, which yield geographically diffuse benefits but carry localized costs will result in resistance from nearby residents who would bear the burden of those costs (Davis and Lester 1988; Krause et al. 2014). Furthermore, we find that 14% of respondents support the deployment of CCS to tackle climate change but only when facilities are proposed for areas elsewhere in the UK and not close to their own local communities. Following Krause et al. (2014), we refer to this as a NUMBY (Not Under My Back Yard) reaction. Similar to the NIMBY (Not In My Back Yard) reaction often present in discussions around renewable energy and nuclear energy facilities, NUMBY refers to opposition to CCS facilities that are to be located within an individual’s local area. This difference between high levels of general public support and contested local projects is a critical issue, which has influenced the trajectory of renewable energy (and other energy projects) in Europe, and specifically the UK (Toynbee 2007). In a study conducted in China, Chen et al. (2015) observed a paradox in their findings: over 90% of respondents expressed that they did not object to the development of CCS in China, yet when investigating perceptions of CCS risks, few respondents were willing to support a CCS project near their local community. Krause et al. (2014) observed a similar pattern in Indiana, USA. 80% of respondents expressed general support for CCS, perhaps as a result of the state’s coal-intensive history, yet over a fifth of the initial supporters change to opposition when the hypothetical CCS facility was proposed near their homes or communities. Thus, careful consideration should be given to the location of potential CCS facilities. Interestingly, despite this observed switch from support to opposition in the sample, WTP amongst these individuals is higher. Those who support CCS in general but opposed a CCS site being located near to their home or community had a mean WTP of £130.99. While this is a somewhat surprising result, it is important to remember that these individuals still support the technology in general and therefore can still have significant WTP for the technology. It could be the case that such individuals would be willing to pay more again compared to those who do not express a NUMBY reaction in order for CCS to be implemented further from their local area.

### Mean Willingness to Pay and Environmental Attitudes

We also explore how environmental attitudes influence WTP values for CCS. Respondents were first asked to indicate the extent to which they agree with the statement “I consider myself as someone who is concerned about the environment” to reflect their environmental identity. Respondents agreeing with this statement, and ultimately having a stronger environmental identity, are willing to pay more for the implementation of CCS. Similarly, respondents with stronger environmental values (as indicated by their disagreement with the statement “economic development is more important than environmental protection”, have a higher WTP. Respondents were also asked to state if they are a member of an environmental organisation. We find that this is a significant factor in WTP for the technology. It is important to note that CCS can draw out inconsistent reactions from environmentalists concerned about climate change as on the one hand it is a mitigation measure and reduces CO_2_ emissions, while on the other hand it can be perceived as further locking us into the use of fossil fuels (Carley et al. 2012). Respondents in our sample who feel highly concerned about climate change are willing to pay more for CCS, indicating that they feel the design of CCS to protect the environment outweighs the risk of extending the transition to more sustainable energy systems. In contrast, Kraeusel and Möst (2012) find in Germany the main argument that CCS mitigates climate change is not sufficient in the context of CCS acceptance. Rather, respondents who are concerned about climate change perceive CCS-related risks higher.

Higher WTP values were elicited from respondents who feel that CCS would be an effective way to reduce emissions and therefore tackle climate change. This was also the case for respondents who indicated that they support the implementation of the technology in the UK. Perceived ineffectiveness was determined to be a significant reason why an individual would reject the implementation of CCS in a focus group study by Oltra et al. (2010) in Spain. In this case, CCS is perceived not as a solution to the problem but a reaction to climate change and thus does not make a meaningful contribution to solving climate and energy problems. However, the study also found that some participants expressed the idea of CCS as a ‘bridging’ technology and as a partial contribution as the basis for their approval of its implementation. Thus, given that the term ‘bridging technology’ was used in the present study, it could be that a similar basis for approval was used amongst the sample.

### Policy Implications

Mean WTP is calculated at approximately £96 as a one-off tax contribution. When scaled up to the affected population, which is the total number of taxpayers in the UK (~37 million in 2024, the total WTP for the implementation of CCS was estimated to be £3.55 billion, a cost value that could be entered into a cost benefit analysis for CCS projects. Informing the public about CCS is an important factor in increasing social acceptance of its implementation. Clear and precise information campaigns would prove useful in garnering support for the integration of CCS into the energy mix. However, the way in which this information is framed and presented to the public should be given careful consideration and drive future research. This study has determined there is willingness to pay for carbon capture and storage however it is also right to assume that CCS implies a net saving as well as a net cost. The decision to elicit WTP was made given that the technology is not currently implemented at scale in the UK at the time of writing. Therefore, investment is needed to get projects off the ground before a net saving through energy bills is evident. Future research should explore the willingness to accept (WTA) for implementing CCS wherein a comparison can be made with findings from this study. In addition, this would add to the limited existing research on WTA in stated preference studies and determine the effectiveness of the WTA framework.

## Data Availability

Due to the sensitive nature of the questions asked in this study, survey respondents were assured raw data would remain confidential and would not be shared.
